# The Clinical Observation and Mechanism of Acupuncture on Cancer-Related Fatigue of Breast Cancer Based on “Gut-Brain Axis”: Study Protocol for a Randomized Controlled Trial

**DOI:** 10.1155/2022/8099595

**Published:** 2022-05-07

**Authors:** Zhuan Lv, Yi-Ming Gu, Rui-Dong Liu, Kai-Qi Su, Xiao-Di Ruan, Yi-Niu Chang, Jing Gao, Rui-Qing Li, Lu Fang, Xiao-Dong Feng

**Affiliations:** ^1^Henan University of Chinese Medicine, Zhengzhou Henan, China; ^2^Department of Rehabilitation Center, The First Affiliated Hospital of Henan University of Chinese Medicine, Zhengzhou, Henan, China; ^3^Department of Breast Surgery, The First Affiliated Hospital of Henan University of Chinese Medicine, Zhengzhou, Henan, China

## Abstract

*Background*. Cancer-related fatigue (CRF) is a painful, persistent feeling of physical and cognitive subjective fatigue related to cancer or cancer remedy. The occurrence of CRF may be related to the hypothalamic-pituitary-adrenaline (HPA) axis, inflammatory mediator theory, and gut microbiota. Moreover, acupuncture could play a vital part in the therapy of CRF. The study will evaluate whether acupuncture can improve fatigue symptoms of CRF patients after breast cancer chemotherapy by regulating the gut-brain axis. *Methods/design*. Seventy patients with CRF will be enrolled in this study, with 35 patients randomly assigned to each group. Blood and feces will be collected at the beginning and end of treatment. Piper fatigue scale, KPS score scale, and quality-of-life scale will be used to observe the changes of fatigue symptoms and life quality of CRF patients and to evaluate the effect of acupuncture on CRF. Then, the method of ELISA will be used to explore the regulatory effect of acupuncture on the HPA axis and inflammatory cytokines. Moreover, based on 16SrDNA, the changes of gut microbiota structure and flora of CRF patients will be observed. Ultimately, the correlation analysis of gut microbiota can be related to inflammatory cytokines, HPA axis, and clinical efficacy evaluation. *Discussion*. This study will identify the effect and the mechanism of acupuncture therapy in CRF. And it will offer an alternative treatment modality for the treatment of CRF after chemotherapy for breast cancer. Furthermore, it will also provide the clinical and theoretical bases for the extensive application of acupuncture therapy in tumor rehabilitation. *Trial Status*. Protocol version number and date: V2.0, 6 May 2021. The trial is registered with the Chinese Clinical Trial Registry on 20 June 2021 (trial identifier: ChiCTR2100047510).

## 1. Introduction

Breast cancer is one of the most common malignancies among women in the world, according to the latest global cancer data in 2020 released by the World Health Organization International Agency for Research on Cancer (IARC); the incidence of breast cancer ranks first in the world [1–3]. With the development of breast cancer research and advanced treatment techniques and monitoring methods, the survival time of people with breast cancer was remarkably prolonged. Nevertheless, on account of the cancer illness and treatment process, the majority of breast cancer survivors have a variety of physical and psychological reactions, such as sleep disorders, cancer-related fatigue, and depression [4]. A large number of patients with breast cancer suffered different degrees of tiredness several months after diagnosis. In particular, nearly half of the patients still experienced severe CRF postdiagnosis [5, 6]. The National Comprehensive Cancer Network (NCCN) defines cancer-related fatigue (CRF) as a persistent subjective sensation caused by cancer or cancer remedy that disrupts normal bodily function and seriously affects the patient's physical function and quality of life. In addition, the incidence of CRF occurs in 70% to 100% of cancer patients who receive chemotherapy [7, 8].

For the past few years, many studies have found that the incidence rate of CRF may be connected to the hypothalamic-pituitary-adrenaline (HPA) axis, inflammatory mediator theory, and gut microbiota [9]. The researchers demonstrated that both the tumor and its treatment could activate the proinflammatory cytokine network [10]. At the same time, these inflammatory factors enter the brain in various ways. Then, these factors disrupt the HPA axis, reduce the synthesis and release of cortisol, and ultimately cause fatigue [11]. Moreover, inflammatory cytokines can be used as an indicator of immune function in patients with CRF [12]. Besides, the gut microbiota is involved in the development of CRF, and it can directly influence immune system cytokines, corticotropin-releasing hormone and cortisol (CORT), thereby affecting the HPA axis and brain function [13, 14].

Nonpharmacological treatment of CRF includes exercise, psychological intervention, health education, CBT, sleep improvement, and nutrition counseling. Moreover, drug intervention mainly includes central stimulants. But these measures mostly belong to the symptomatic treatment; the overall effect is not satisfactory.

Given this, we are actively exploring new approaches. Abundant studies have found that traditional Chinese medicine plays a vital part in the therapy of CRF. In particular, acupuncture has the advantages of dredging the channels and collaterals, regulating the viscera, running qi and blood, strengthening the body, and dispelling the harmful factors. In the recent study, it is clear that acupuncture can regulate the changes of CORT and adrenocorticotrophic hormone (ACTH) levels in the HPA axis by affecting the changes of gut microbiota and inhibiting the activation of inflammatory cytokines, and ultimately reduce the level of cancer-related fatigue in patients. This way provides a theoretical basis for the extensive application of traditional acupuncture in tumor rehabilitation. Meanwhile, it has the advantages of low cost, short time consumption, and fewer side effects. Therefore, the study designed a randomized controlled trial comparing acupuncture and rehabilitation exercise to observe the clinical efficacy of acupuncture in the treatment of CRF patients with breast cancer after chemotherapy. Our purpose is to gain further results through laboratory examinations and scale assessments. These results will help to demonstrate whether acupuncture can improve CRF as an effective new treatment method in these patients.

## 2. Objectives

1. To observe the treatment of acupuncture in improving CRF with breast cancer after chemotherapy in patients
2. To clarify that the acupuncture affects the expression level of inflammatory cytokines in the immune system by regulating the structural changes of intestinal flora, then regulates the changes of HPA axis function, and finally plays a role in improving fatigue symptoms

## 3. Design and Methods

### 3.1. Trial Design and Setting

**Figure 1:**
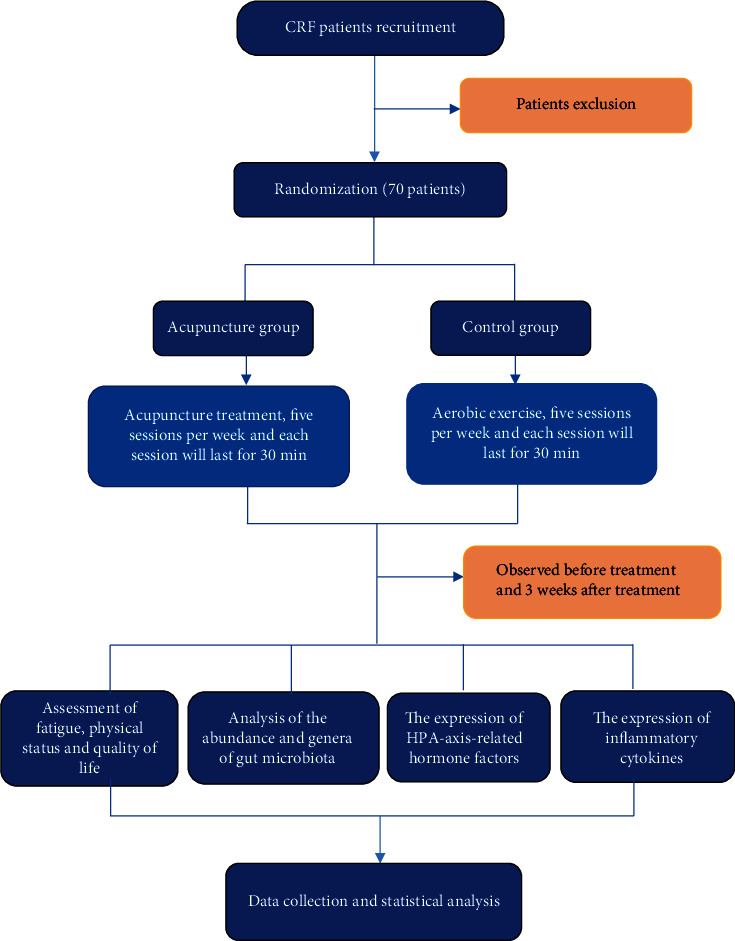
Flowchart of trial procedures.

This is a randomized controlled study to explore whether acupuncture can improve fatigue symptoms of CRF patients after breast cancer chemotherapy by regulating the gut-brain axis. Seventy participants will be randomly assigned to either the acupuncture group or the primary treatment group with 35 patients in each group in a 1 : 1 ratio. Relevant tests will be tested on both groups at the baseline and after 3 weeks of the treatment. Blood and feces will be collected at the beginning and end of treatment. Piper fatigue scale, KPS score scale, and quality-of-life scale will be used to observe the changes in fatigue symptoms and quality of life of CRF patients in two groups before and after treatment and to inspect the curative effect of acupuncture on CRF. Then, the method of ELISA will be used to explore the regulatory effect of acupuncture on the HPA axis and inflammatory cytokines. Moreover, based on 16SrDNA technology, the changes in gut microbiota structure and flora of CRF patients will be observed. Ultimately, the correlation analysis of gut microbiota content can be related to inflammatory cytokines, HPA axis, and clinical efficacy evaluation (Figure 1 and Table 1).

### 3.2. Participants

The participants with breast cancer after chemotherapy who suffer from fatigue according to the diagnostic criteria of CRF will be collected from the First Affiliated Hospital of Henan University of Chinese Medicine. All participants will be assessed with various tests and scales at the baseline. If the patients meet the inclusion criteria, they will sign the informed consent to prepare for the next trial.

### 3.3. Eligibility Criteria

#### 3.3.1. Inclusion Criteria

Inclusion criteria require that the participants (1) match the diagnosis of having CRF according to the diagnostic criteria of ICD-10 for CRF; (2) with breast cancer will be diagnosed by pathology or cytology; (3) must be between 18 and 80 years; (4) must have a KPS score above 50 and have an expected survival time of half a year or more; (5) have previously undergone chemotherapy, and they will have no other treatment plan for the next 21 days; (6) have no mental disorder or cognitive impairment and have normal language expression ability and can understand the scale content and cooperate with treatment; (7) have no serious heart, liver, or kidney diseases; and (8) have signed a written informed consent form.

#### 3.3.2. Exclusion Criteria

Participants will be excluded if they (1) have severe systemic diseases, such as cardiovascular disease, acute infectious disease, liver or kidney diseases; (2) have mental or psychological diseases with definite diagnosis, including cognitive impairment, anxiety, and depression syndrome; (3) are pregnant and breastfeeding; (4) are allergic to alcohol and needles or afraid of the acupuncture; (5) have a language communication disorder or other reasons cannot allow them to cooperate with follow-up and examination; and (6) have another clinical trial during enrolment.

### 3.4. Randomization and Blinding

In this study, the random numbers will be created by SAS (Version 9.3) (SAS Institute Inc., Cary, NC, USA). A nonparticipating statistician will manipulate the random numbers, who will inform the therapists of each randomized outcome via text message.

At the same time, depending on the particularity of acupuncture treatment, it is difficult to realize double-blind. In order to follow the principle of the blind method, the separation of the operator and statistician is strictly implemented throughout the whole research process. Firstly, when we start treating patients, all CRF participants do not know their grouping situation, and the operator selects corresponding treatment options according to a different grouping of subjects for treatment. Secondly, the data on gut microbiota in this study will be statistically analyzed by a third party, and the statistician will be unaware of the grouping and its clinical significance.

### 3.5. Sample Size

According to the results of previous literature (clinical study on an acupuncture fatigue group for cancer-related fatigue of qi and blood deficiency type), the average fatigue scale score of the control group was 5.17 ± 1.26, and the average score of the fatigue scale in the acupuncture group was 4.17 ± 1.14. In our study, we will set the following parameters: *α* = 0.05, 2-sided, power = 0.9. Our software PASS (Version 15.0) will be used to calculate the sample size of the treatment group and the control group, and the result is 30 in each group. Considering the loss of sample size caused by the 15% dropout rate, the final calculation will require at least 35 cases in the treatment group and 35 cases in the control group, and a total of at least 70 cases will be included.

### 3.6. Intervention

The different treatments will be performed for thirty-five participants in each group. They will have experienced it five times a week for three weeks in separate compartments. These therapists are experienced doctors who have obtained medical qualifications. Before the trial, they will receive training to ensure adherence to the study protocol. Meanwhile, each therapist will be kept ignorant of the group allocation. The various separate interventions in two groups are as follows.

In the treatment group, according to the relevant literature research, the most commonly used acupoints for the treatment of CRF were summarized, and Zusanli (ST36), Sanyinjiao (SP6), Qihai (CV6), Guanyuan (CV4), and Baihui (GV20) will be selected. The orientation of acupoints derives from the planning textbook of traditional Chinese medicine colleges and universities, *Acupuncture and Moxibustion Science*. First, in the study, the therapists will use the single-use sterile needles (Hwato, Suzhou, China) to puncture these patients. 40 mm long needles (0.35 mm in diameter) are used to puncture points on the limb and abdomen. 25 mm long needles (0.3 mm in diameter) are used for head points. All needles are manually manipulated by flat reinforcing and flat reducing methods to create the certain experience termed as De Qi that occurs in both the patients and therapists. Participants will receive five acupuncture sessions per week, and each session will last for 30 min. In the control group, participants will take on aerobic exercise through jogging mode, in which the heart rate will be controlled at 50% of the maximum heart rate (maximum heart rate = 220 − patient′s age). They will also receive five exercise sessions per week, and each session will last for 30 min.

### 3.7. Primary Outcomes

We have three primary outcomes for this study: assessing the improvement of fatigue, physical performance, and the quality of life in CRF patients before treatment and 3 weeks after treatment. All evaluations will be performed by two professional physicians. They will be blinded to the treatment allocation.

The Piper fatigue scale will be used to evaluate the severity of fatigue before treatment and 3 weeks after treatment. It uses a 10-point scale of numerical descriptions, including nine items. Each item is rated on a scale of 0 to 10, with 0 indicating no fatigue and 10 indicating the most severe fatigue. In addition, 1 to 3 is for mild fatigue, 4 to 6 for moderate fatigue, and 7 to 10 for severe fatigue, and the fatigue integral is the total divided by 9. Overall, the higher the scores get, the more tired patients feel.

The KPS rating scale will be used to evaluate the body function status of patients before and 3 weeks after treatment. The KPS score is the functional status rating criterion. And the higher the KPS score gets, the better the health and physical status will be. Besides, it is generally believed that the KPS score above 80 is independent, 50-70 is divided into the semi-independent level, and below 50 is a dependent score, meaning someone needs help in their life. Patients with scores greater than 80 will have better postoperative status and longer survival time.

The EORTC core quality-of-life scale will be used to evaluate patients' quality of life before treatment and 3 weeks after treatment. QLQ-C30 (V3.0) is the most widely used method in the world to measure the quality of life of cancer patients. The inventory consists of 30 questions, from five kinds of function (physiology, daily life, cognitive, emotional, and social functions), three kinds of symptoms (fatigue, pain, nausea, and vomiting) and overall health status, the overall quality of life, and another separate six (sleep, appetite, diarrhea, constipation, difficulty breathing, and economic status) to measure the patient's quality of life. All the measurements were combined to get a score, which was proportional to the quality of life of the patients.

### 3.8. Other Outcome Measures

#### 3.8.1. The Expression of HPA Axis-Related Hormone Factors and Inflammatory Cytokines

Blood samples of CRF patients in the acupuncture group and the basic treatment group will be collected for testing before and 3 weeks after treatment, respectively. Fast-fasting blood samples will be collected from all subjects in the morning. And the collected samples will be centrifuged at 3000 rpm at 4°C for 15 min. Moreover, Serums will be collected and stored at -80°C. Human serum CORT, ACTH, IL-6, IL-1*β*, and TNF-*α* enzyme-linked immunosorbent assay kit will be used for detection. In addition, the ELISA kit will be balanced at room temperature for 30 min, and the samples will be dissolved at room temperature. Standard samples with different gradient concentrations will be prepared, and a standard diluent will be added. We will add 50 *μ*l of biotin-binding antibody to each well, seal the plates, place in a horizontal shaker to shake slightly and mix, and incubate at room temperature for 2.5 h. We will discard the supernatant, add the detection antibody, and incubate at room temperature for 1 h. We will discard the supernatant and incubate in the dark at room temperature. Then, we will add the stop solution and, after fully mixing, detect the absorbance OD value at 450 nm with the enzyme plate analyzer.

#### 3.8.2. The Changes in Gut Microbiota

Fecal specimens will be collected before and 3 weeks after treatment for detection. The subjects should collect the feces immediately after defecating in the morning to ensure the feces are fresh. Then, the central part of the feces should be collected by using sterile cotton swabs, and contact with the feces should be avoided. The amount of feces should be collected as much as possible. Subsequently, we will put fresh feces in a sterile feces box and immediately put the feces box in a -80°C refrigerator for preservation. The feces will be taken out of the refrigerator and placed on ice. 200 mg of feces will be taken out and put into a sterile centrifuge tube. The DNA of gut microbiota from the feces samples will be extracted, and the purity of DNA will be measured by the A260/280 method. Then, primers will be found according to 16SrDNA sequences of gut microbiota, and a BLAST GenBank will be used to compare the specificity of the primers for PCR amplification. Subsequently, Gel recovery, separation, and purification of DNA amplification products of gut microbiota will be performed according to the instructions of the QIAEX II Gel Extraction and Purification Kit operation manual. A PE library will be constructed for Illumina sequencing. Finally, the gut microbiota of patients in each group will be analyzed, including optimizing sequence information statistics and OUT analysis, counting alpha diversity index of samples, drawing dilution curve, and analyzing alpha diversity, PCoA, community composition analysis, and species difference analysis.

### 3.9. Informed Consent and Safety Assessments

All patients who have signed the written informed consent will receive appropriate treatment. Physical examinations and vital sign testing of participants are required before, during, and after treatment. Moreover, there may be some adverse reactions after acupuncture, but they are rare and slight. Acupuncture sickness may occur because of patients' physical problems or emotional stress, which can be relieved after stopping acupuncture and moxibustion and taking proper rest. Hemorrhage, hematoma, and other phenomena may occur after acupuncture, which can disappear after local pressing. Finally, during the acupuncture process, the doctor will strictly disinfect the operation. Adverse events will be recorded in detail for safety evaluation and submitted to the institutional review board (IRB).

### 3.10. Quality Control, Data Management, and Monitoring

Ahead of the recruitment and treatment of this study, the whole research team will be requested to learn in professional training classes, which guarantees the smooth progress of the plan and the sufficient understanding of the management procedure of the trial. The case report forms will preserve the treatment plans, crucial adverse reactions, and safety assessments of each patient throughout the whole process of the protocol. Besides, the case report forms will be maintained by our dedicated data management team and will be kept in a closed environment with security and access restrictions. Our research team members will ensure that all data are confidential and prevent data leakage and loss. The patients' information and records will be preserved on password-protected servers. Only the research team members are allowed to access patient information and perform data quality control. The data quality will be checked regularly by the research assistant under the monitors. The Ethics Committee of the Henan University of Chinese Medicine will monitor the data, supervise the trial, audit the trial every 3 months, and manage the progress and standardization of the trial.

### 3.11. Statistical Analysis

Data analysis will be conducted by using the IBM SPSS for Windows (Version 21.0). Basic descriptive statistics (mean and SD ($\overset{¯}{x}\pm s$)) will be reported for the characteristics and outcomes of each participant. The statistical significance is defined as *P* < 0.05 for all statistical analyses.

The Piper fatigue scale, KPS score, quality-of-life score, expression levels of HPA axis-related factors and inflammatory cytokines, and DNA copy number of gut microbiota are all measurement data. When comparing between groups, if the normal test and homogeneity test of variance are met, the *t*-test of two independent samples will be used. If it meets the normal test but does not meet the homogeneity test of variance, two independent sample-corrected *t-*tests will be used. If the normal test is not met, the Wilcoxon Man-Whitney *U* rank sum test for comparison between the two samples will be used.

The correlation analysis between gut microbiota and clinical efficacy score, HPA axis-related factors, and inflammatory cytokines will be performed using Pearson correlation analysis (for data obeying normal distribution) or Spearman correlation analysis (for data not obeying normal distribution).

## 4. Discussion

We proposed a randomized controlled trial to determine whether acupuncture can regulate the changes of CORT and ACTH levels in the HPA axis by affecting the changes of gut microbiota and inhibiting the activation of inflammatory cytokines, and ultimately reduce the level of cancer-related fatigue in patients. If acupuncture is effective in treating CRF, it will offer a novel therapeutic method for wider application in tumor rehabilitation.

The occurrence of CRF is related to the tumor itself, the treatment measures for the tumor, the complications of the tumor, and the related psychosocial factors [9]. And its pathogenesis may be related to the inflammatory mediator theory and HPA axis. Abundant studies have confirmed that changes in the HPA axis play a vital part in the occurrence and development of fatigue [15]. The HPA axis includes CORT, corticotropin-releasing hormone (CRH), and ACTH [16]. In addition, a randomized controlled trial found that the ACTH levels in patients' serum with CRF were obviously higher than those in the nonfatigue group, and the expression of ACTH was highly positively correlated with CRF [17]. Weinrib et al. found that the expression of CRH in the serum of ovarian cancer patients was negatively correlated with CRF [18]. McEwen et al. have shown that the HPA axis regulates the levels of proinflammatory cytokines by regulating glucocorticoid changes [19]. CORT is a glucocorticoid, which is mainly regulated by adrenocorticotropic hormone, and it can also regulate the level of the CORT hormone. However, antitumor therapy can disrupt this feedback regulation, causing the level of cortisol to be unregulated, leading to the occurrence of CRF.

In addition, relative animal experiments and clinical trials have confirmed that tumor-related therapy can lead to the decrease of immune function, and the occurrence of CRF is closely related to the body immune state. At present, inflammatory cytokines, T lymphocyte subsets, and immunoglobulin are the indicators reflecting the immune function of CRF patients [20]. Among them, inflammatory cytokines are widely used in clinical practice to reflect the immune state in vivo. For example, IL-6 is secreted by macrophages and monocytes, and it can stimulate the proliferation and differentiation of B lymphocytes. Meanwhile, IL-1*β* plays a central role in immune regulation and inflammatory responses, and it is associated with many immunological diseases. In addition, TNF-*α* is secreted by macrophages, mast cells, and NK cells, and it mainly stimulates macrophages to release the inflammatory cytokines and prostaglandin-related inflammatory mediators [21, 22].

The gut-brain axis is composed of the central nervous system, the neuroendocrine system, the neuroimmune system, the HPA axis, the autonomic nervous system, and the gut microbiota [23, 24]. Gut microbiota is a complex microbial community in the human gut; it plays a crucial part not only in the occurrence and progression of tumors but also in the adverse reactions caused by tumor therapy [14]. Changes in gut microbiota can affect the occurrence process of CRF, and CRF can also cause changes in the abundance of gut microbiota [25]. In addition, gut microbiota and its metabolites may influence the progression of neuropathic diseases by participating in the regulation of nervous system function through the gut-brain axis. Similarly, the nervous system influences the composition and quantity of gut microbiota through the gut-brain axis. The immune system is an important pathway between the gut microbiota and the brain. At the same time, gut microbiota can directly influence immune system cytokines, CRH and CORT, thereby affecting the HPA axis and thus brain function [26, 27].

CRF belongs to the category of “deficiency of fatigue” in traditional Chinese medicine. Meanwhile, the main pathogenesis of CRF is deficiency of qi, blood, and yin and yang [28]. Furthermore, the syndrome is mainly characterized by deficiency of qi and blood, disharmony between the liver and stomach, stagnation of qi and blood stasis, deficiency of yin and fire, and condensation of phlegm and dampness [29, 30]. Traditional Chinese medicine (TCM) plays an important role in improving CRF, including single herbs, prescriptions, proprietary Chinese medicine products, and aqueous solutions made from herbal extracts, whereas acupuncture has its unique advantages in the treatment of CRF. It can stimulate acupoints through filiform needles, apply the technique of reinforcing and reducing energy to stimulate meridians and qi, and then play the role of dredging meridians, regulating viscera, running qi and blood, and strengthening and dispelling pathogenic factors [31]. Studies have shown that acupuncture is effective in improving the complications of cancer, such as pain, insomnia, depression, nausea, vomiting, myelosuppression, and fatigue [32]. A clinical trial found that acupuncture at Zusanli (ST36), Hegu (LI4), Qihai (CV6), Sanyinjiao (SP6), and Guanyuan (CV4) improved fatigue and quality of life in patients with lung cancer [33]. In addition, a meta-analysis found that the most frequently used acupoints in the treatment of CRF were Zusanli (ST36), Guanyuan (CV4), Qihai (CV6), Sanyinjiao (SP6), Hegu (LI4), and Baihui (GV20) [34]. Some studies have found that acupuncture can regulate the synthesis, secretion, and biological activity of cytokines IL-2, IL-6, and TNF-*α* in vivo, thus alleviating CRF [35]. At the same time, acupuncture can effectively regulate the disturbance of gut microbiota, restore the microecological balance of the body, effectively regulate the function and structure of gut microbiota at different acupoints, and improve the proportion and diversity of bacterial flora [36].

Therefore, we will choose Zusanli (ST36), Sanyinjiao (SP6), Guanyuan (CV4), Qihai (CV6), and Baihui (GV20) acupuncture points to perform acupuncture treatment on CRF patients after breast cancer chemotherapy. In our study, we will confirm that acupuncture therapy can reduce fatigue, improve the physical status, and improve the quality of life in CRF patients after chemotherapy for breast cancer. Moreover, acupuncture treatment will lead to changes in the HPA axis, and the expression level of ACTH in the serum is lower than that in the control group, while the expression level of CORT is higher than that in the control group. In addition, acupuncture treatment will reduce the expression levels of inflammatory cytokines IL-1*β*, IL-6, and TNF-*α* in the serum and further identify its effect on inflammatory cytokines and immune regulation. Finally, the changes in gut microbiota structure and genus in the acupuncture group and the control group will be observed, and further correlation analysis will be conducted with the HPA axis, inflammatory cytokines, and clinical efficacy evaluation. In all, acupuncture therapy will regulate the changes of CORT and ACTH hormone levels in the HPA axis by affecting the changes of gut microbiota and inhibiting the activation of inflammatory cytokines, and ultimately reduce the level of cancer-related fatigue in breast cancer patients.

If the primary hypothesis of this study is proven, acupuncture treatment has the potential to be used for reducing fatigue sensation and improving the quality of life of patients with breast cancer. Acupuncture therapy of traditional Chinese medicine would offer an alternative treatment modality and objective evidence for the treatment of CRF after chemotherapy for breast cancer and then alleviate the economic burden on patients and the society. Ultimately, the proposed study will also provide the clinical and theoretical bases for its extensive application of acupuncture therapy in tumor rehabilitation.

## Figures and Tables

**Table 1:** Schedule of enrolment, interventions, and assessments.

	Enrolment	Allocation	Postallocation			Closeout
Timepoint	Week -1	Week 0	Week 1	Week 2	Week 3	Week 4
Enrolment						
Eligibility screen	X					
Informed consent	X					
Randomization		X				
Interventions						
Group A			X	X	X	
Group B			X	X	X	
Assessments						
Piper fatigue scale		X				X
KPS rating scale		X				X
EORTC-QLQ–C30		X				X
Adverse events			X	X	X	X
Blood detection		X				X
Feces detection		X				X

## Data Availability

The data on the study will be available from the corresponding author upon request.

## Abbreviations

CRF: Cancer-related fatigue
HPA: Hypothalamic-pituitary-adrenaline
IARC: The World Health Organization International Agency for Research on Cancer
NCCN: National Comprehensive Cancer Network
CORT: Cortisol
CBT: Cognitive behavioral therapy
ACTH: Adrenocorticotropin-releasing hormone
ICD: International Classification of Diseases
CRH: Corticotropin-releasing hormone
TCM: Traditional Chinese medicine
PCoA: Principal coordinate analysis.

## Additional Points

*Protocol Amendments*. The principal investigator will notify the corresponding office. Then, they will submit a copy of the amended paper to the institutional review board for investigation and storage in the archives. Meanwhile, our group will again reserve the renewal protocol. *Dissemination Policy*. The final outcomes of the research report will be submitted to the Henan Administration of Traditional Chinese Medicine. It is planned to share the results in a peer-reviewed journal, forms of brochures, and conferences.

## Supplementary Materials

Supplementary Materials SPIRIT 2013 checklist: recommended items to address in a clinical trial protocol and related documents.
