# Supplementary figures and images for: The Clinical Observation and Mechanism of Acupuncture on Cancer-Related Fatigue of Breast Cancer Based on “Gut-Brain Axis”: Study Protocol for a Randomized Controlled Trial
Source: Dis Markers. 2022 May 7;2022:8099595. doi: 10.1155/2022/8099595 (PMC9107368; doi:10.1155/2022/8099595)

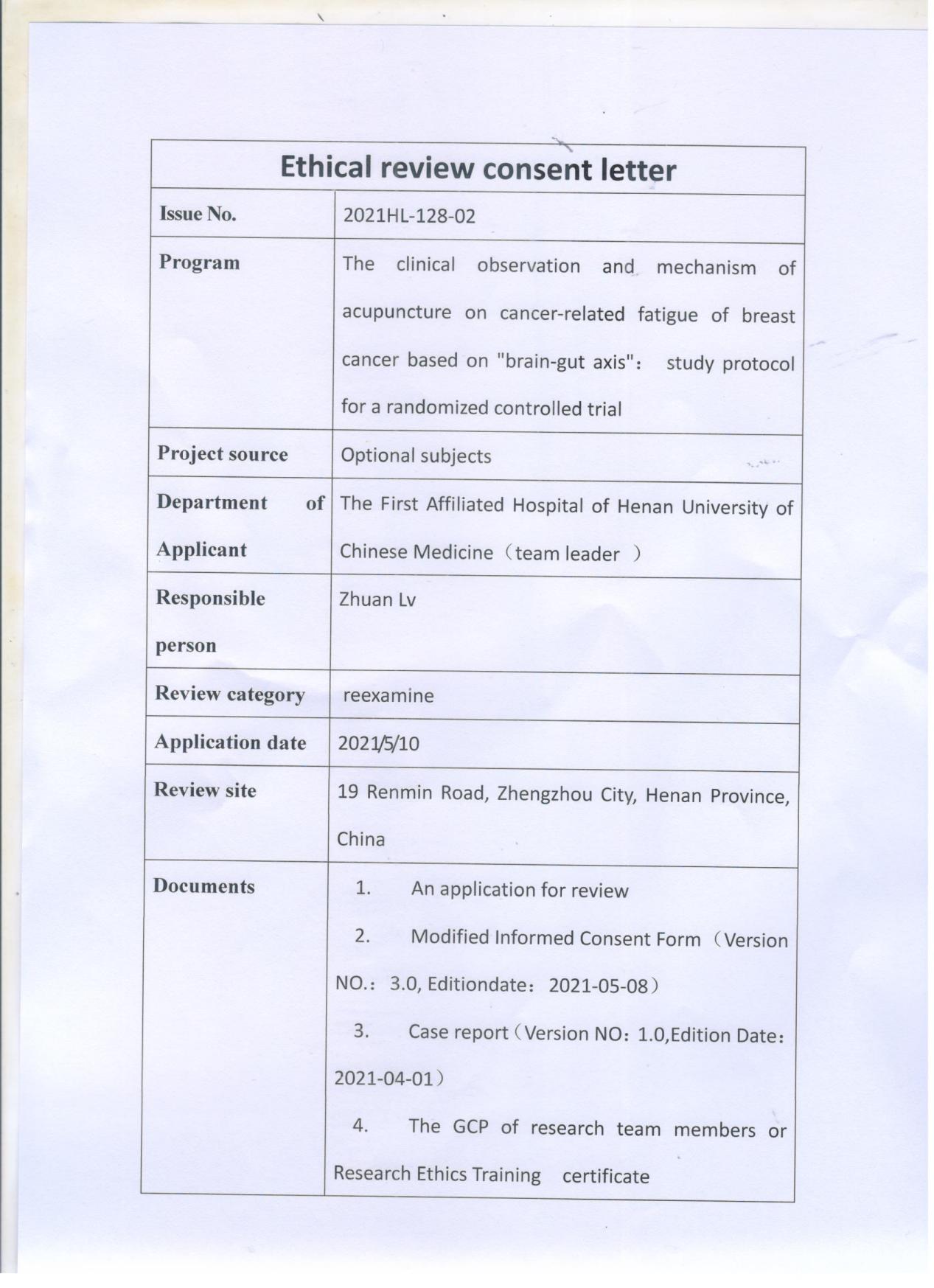


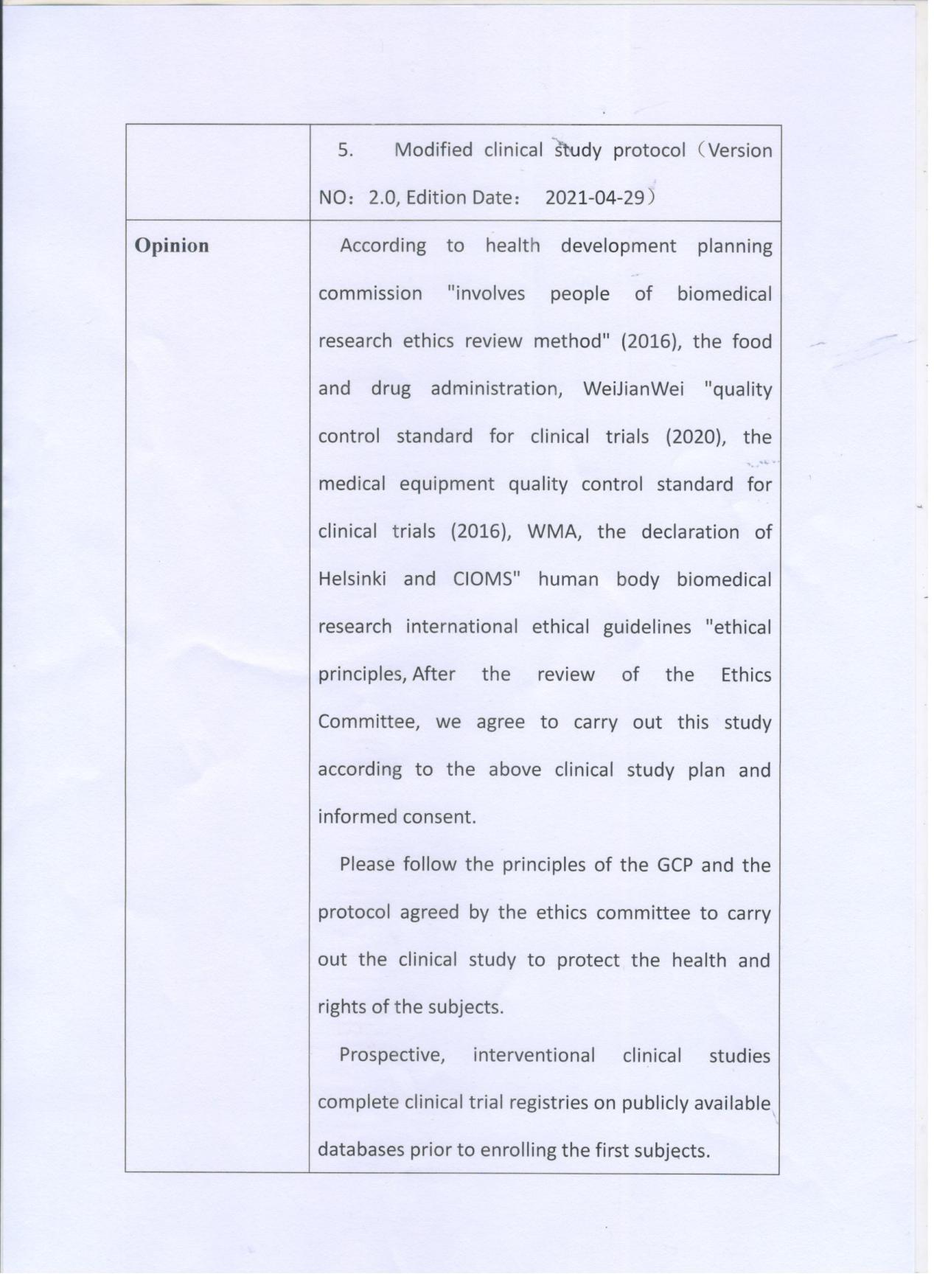


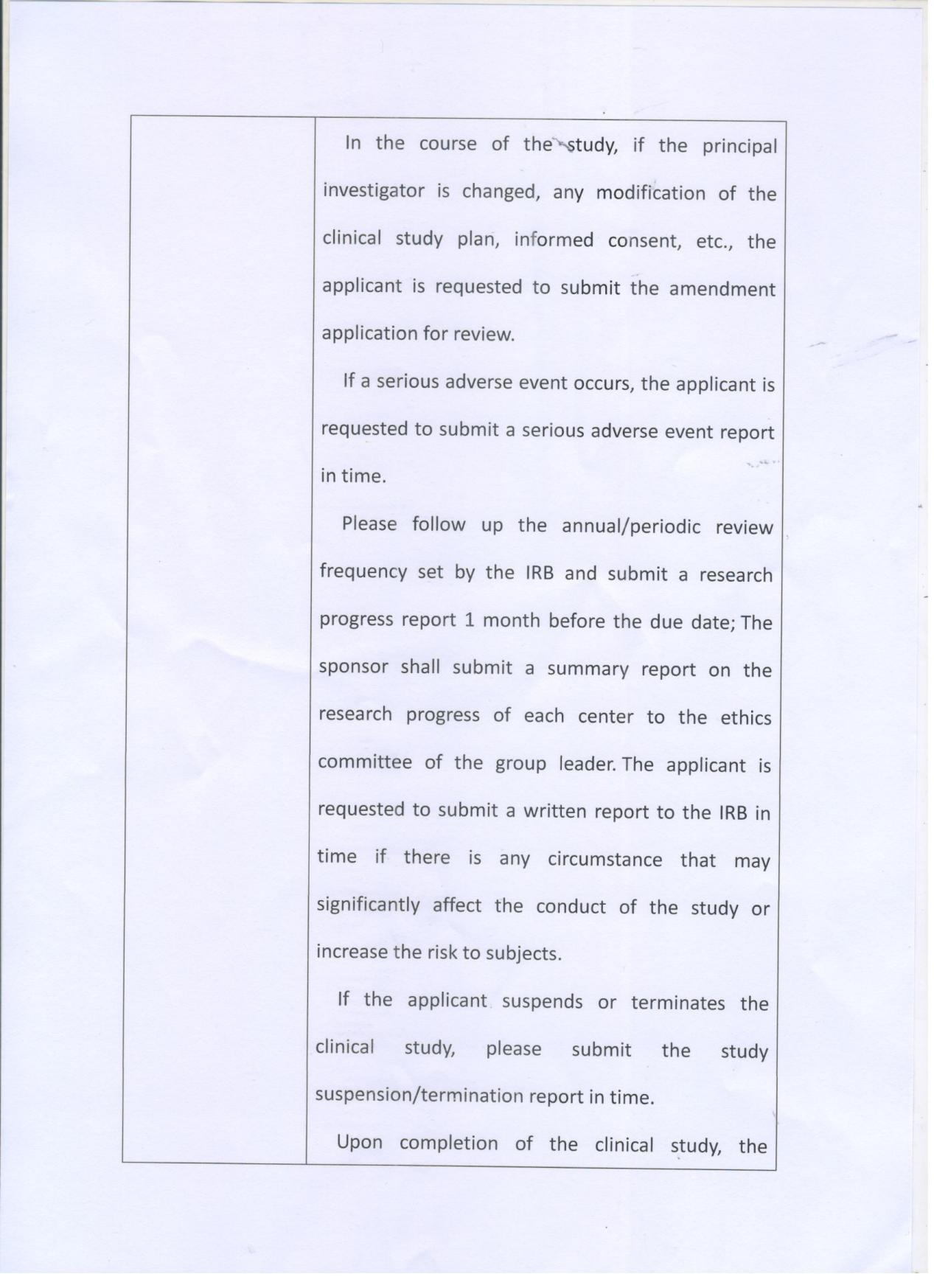


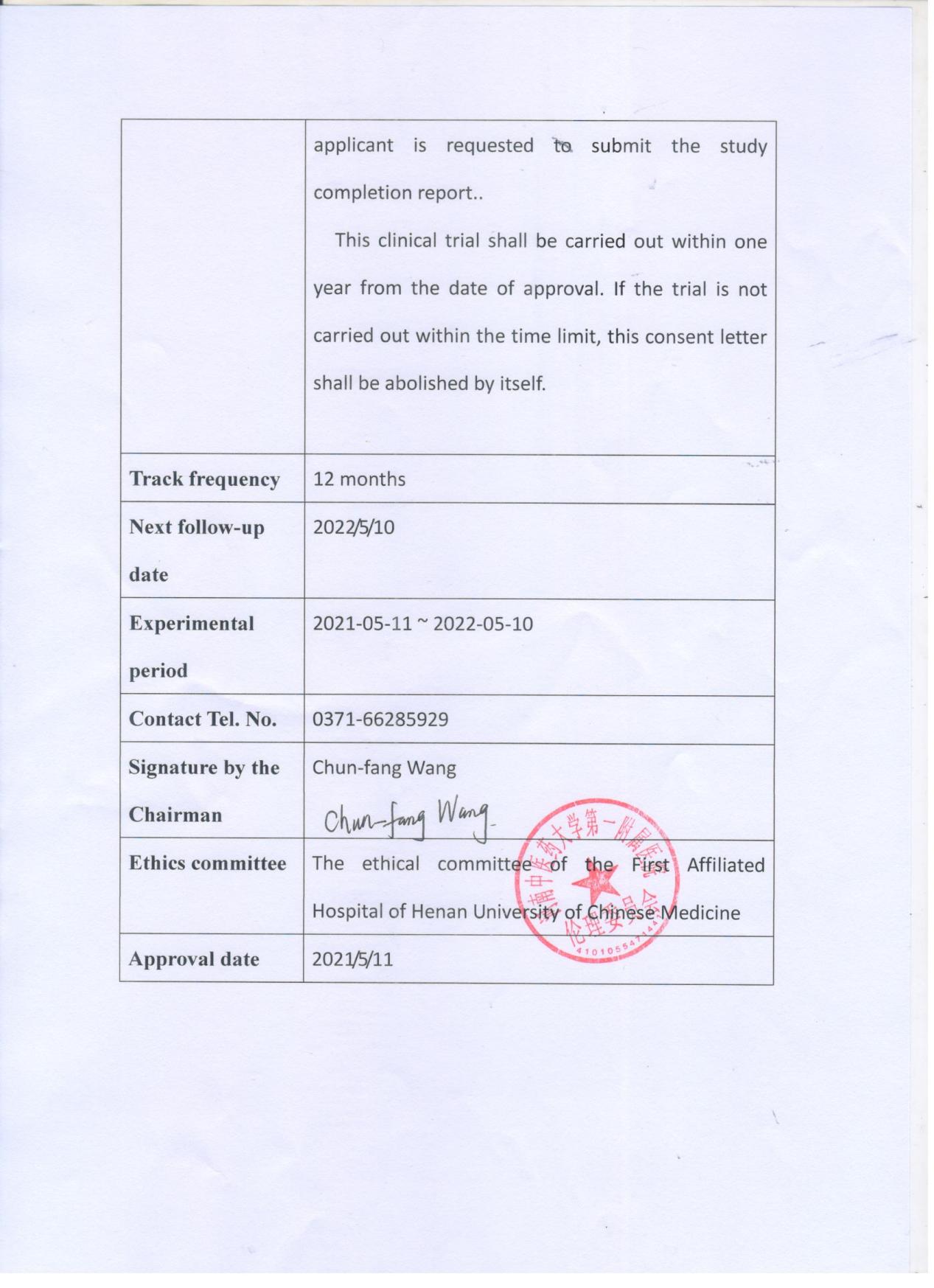


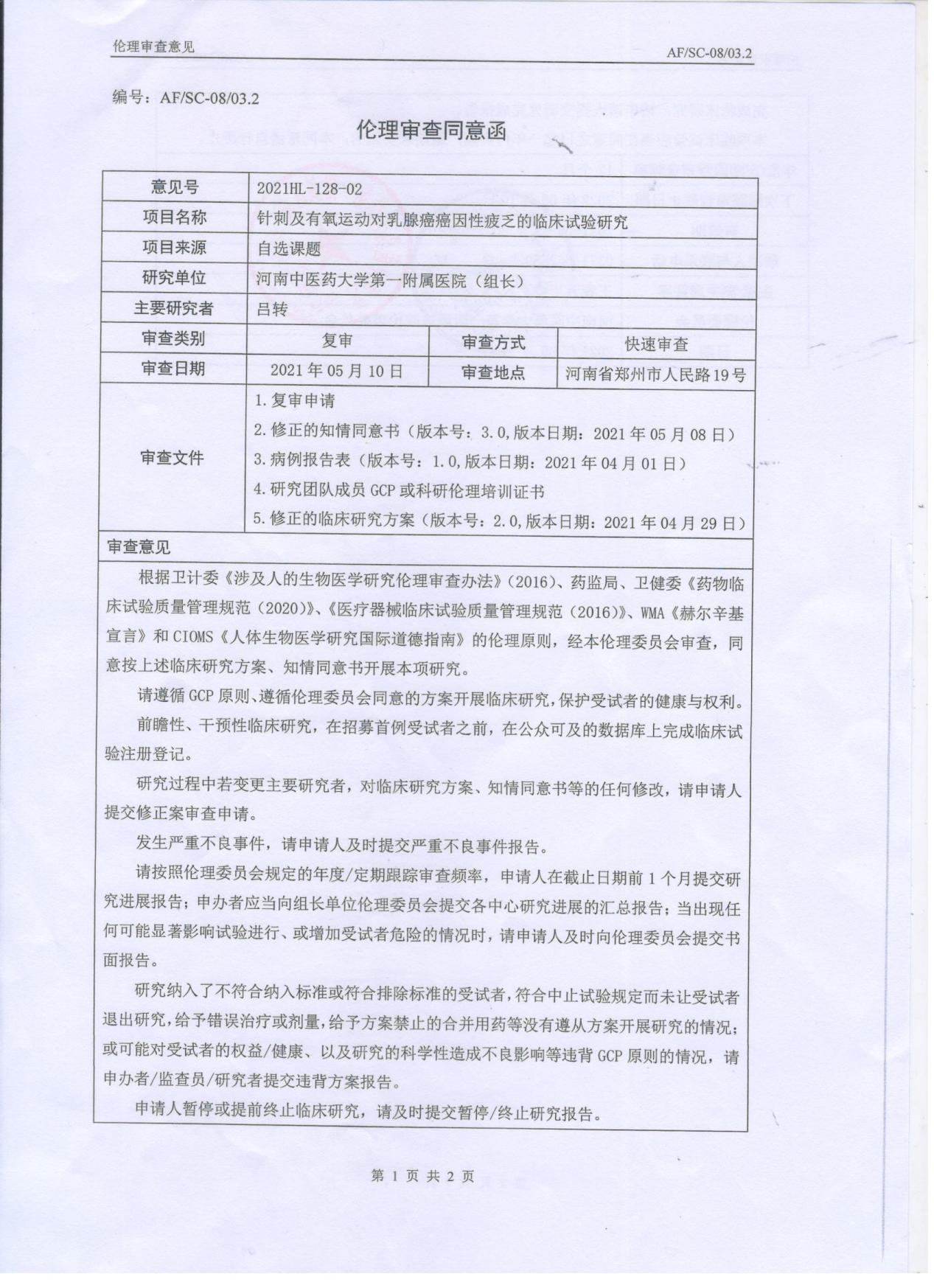


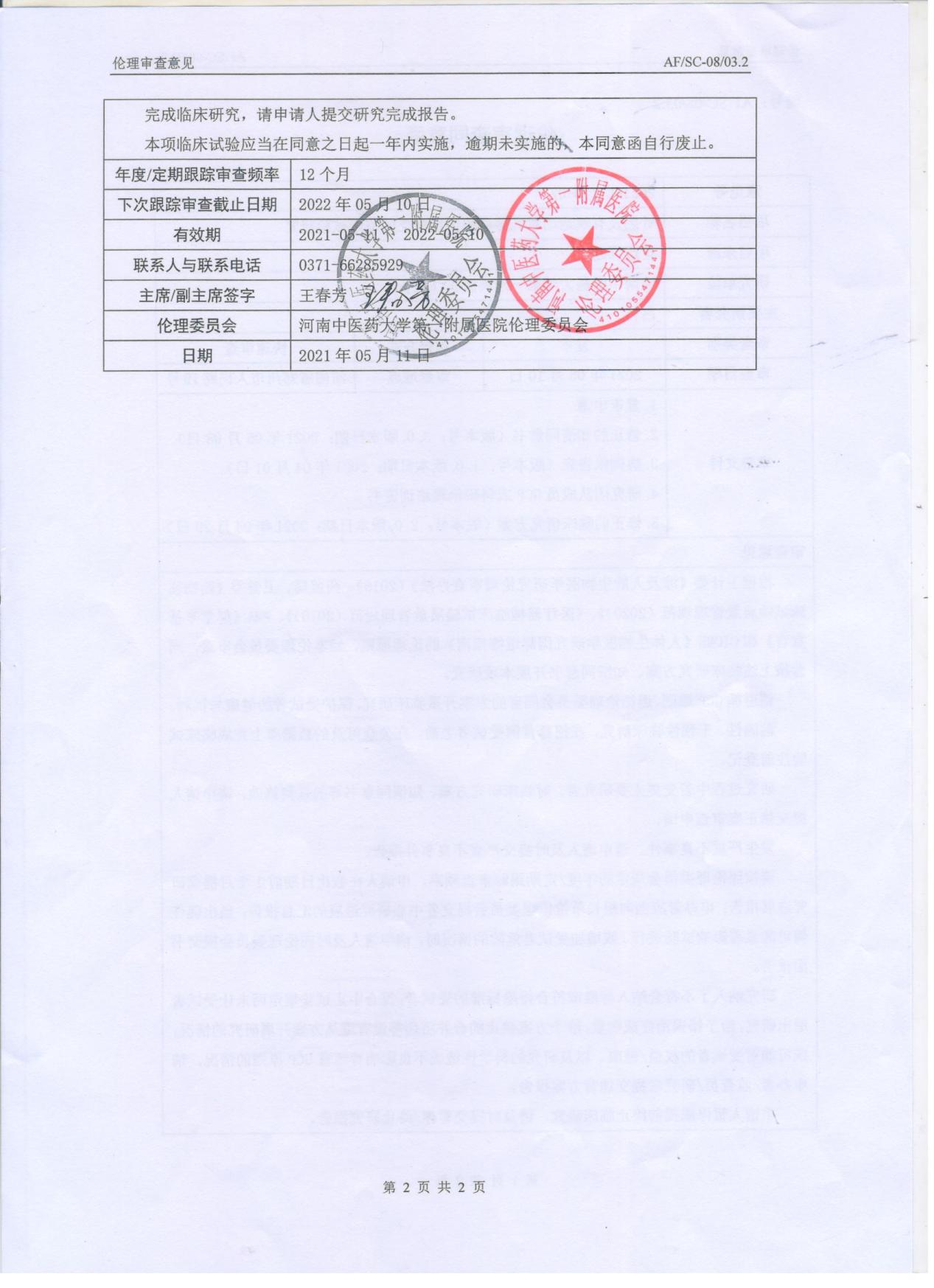

Supplement: Supplementary Materials — SPIRIT 2013 checklist: recommended items to address in a clinical trial protocol and related documents. [file 8099595.f1.zip › new supplementary material 3 Ethical review consent.docx]
