# Supplementary material for: The Clinical Observation and Mechanism of Acupuncture on Cancer-Related Fatigue of Breast Cancer Based on “Gut-Brain Axis”: Study Protocol for a Randomized Controlled Trial
Source: Dis Markers. 2022 May 7;2022:8099595. doi: 10.1155/2022/8099595 (PMC9107368; doi:10.1155/2022/8099595)
Supplement: Supplementary Materials — SPIRIT 2013 checklist: recommended items to address in a clinical trial protocol and related documents. [file 8099595.f1.zip › new supplementary material 2 Informed consent.doc]

**Informed consent**

Dear participants

Your doctor has diagnosed you with a cancer-induced fatigue disease.

We will invite you to participate in a clinical trial research project based on the brain-gut axis to explore the clinical and mechanistic study of acupuncture on breast cancer cancer fatigue.This study started with the TCM acupuncture treatment, discussed the clinical efficacy evaluation of fatigue, survival quality and physical strength in CRF patients after breast cancer chemotherapy, and investigated the mechanism of acupuncture on CRF after breast cancer chemotherapy based on the influence of intestinal flora on the HPA axis.Before you decide whether to participate in this study, to help you understand the study and why the study, the procedures and duration of the study, the benefits, risks and discomfort that may bring to you after participating in the study.If you wish, you can also discuss it with your relatives and friends, or ask your doctor to give an explanation to help you make a decision.

**Introduction**

1.Why is this study being conducted?

Cancer-Related Fatigue (CRF) is a group of symptoms in cancer patients with subjective sensory fatigue, which was first proposed by Haylock and Hart scholars.Tumor patients with fatigue is a typical physical and mental illness, it not only makes the lack of physical feeling strength, also affect the patient's inner emotional world and cognitive response to the outside world, seriously reduce the quality of life of patients, increased the fear of disease and treatment, weakened the compliance, and even forced patients refused to accept treatment without reason.This disease makes the patient physical and mental injury, even after sleeping, relaxation, rest and other common measures to relieve fatigue, the symptoms still can not be relieved.The NCCN guidelines define CRF as "a subjective sense of fatigue involving physiological, emotional, and cognitive levels associated with tumor or tumor treatment".The disease is very common in tumor patients and can occur at any stage of the tumor course.According to the statistics, about 82% -96% of the patients receiving radiotherapy will develop CRF, and the proportion of the patients receiving chemotherapy is 65% -100%.At present, CRF is divided into two categories: drug-based intervention, including central stimulant, and non-drug intervention, including physical activity and psychosocial intervention.Most of these measures are symptomatic treatment, and the overall curative effect is not satisfactory.

In view of this, actively exploring new methods and studying the optimal treatment plan is the key to the current treatment of cancer fatigue.Therefore, this study, from the analysis of TCM acupuncture therapy, stimulates the human meridians and blood, improve the human physiological function, so as to reduce or eliminate the fatigue state of breast cancer patients, comprehensively improve the physical condition of patients, improve the quality of life of patients, and find its effective mechanism.

This study will be conducted in the outpatient clinic of the Rehabilitation Center of the First Affiliated Hospital of Henan University of Chinese Medicine. It is expected that 70 subjects with chemotherapy for breast cancer cancer fatigue will participate voluntarily.

This study has been agreed upon by the research administration.The Ethics Committee of the First Affiliated Hospital of Henan University of Chinese Medicine has considered the study in accordance with the principles of the Declaration of Helsinki and in line with medical ethics.

2. Who is suitable to participate in the study?

Inclusion criteria require that the participants (1) match the diagnosed with CRF according to the diagnostic criteria of ICD-10 for CRF; (2) with breast cancer will be diagnosed by pathology or cytology; (3) must be between 18–80 years; (4) must have a KPS score above 50, and they will be expected survival time of half a year or more; (5) have previously undergone chemotherapy, and they will have no other treatment plan for the next 21 days; (6) have no mental disorder or cognitive impairment, and have normal language expression ability, and can understand the scale content and cooperate with treatment; (7) have no serious heart, liver or kidney diseases; (8) have signed a written informed consent form.

***Exclusion criteria***

Participants will be excluded if they (1) have severe systemic diseases, such as cardiovascular disease, acute infectious disease, liver or kidney diseases; (2) have mental or psychological diseases with definite diagnosis, including cognitive impairment, anxiety and depression syndrome; (3) are pregnant and breastfeeding; (4) are language communication disorder or other reasons cannot cooperate with follow-up and examination; (5) are allergic to alcohol and needles or afraid of the acupuncture.

**3.What will need to be done if participating in the study?**

(1)Before you are enrolled in the study, you will undergo the following checks to determine if you can participate in the study:

The doctor will ask, record your medical history, and give you a comprehensive physical and psychological examination.You need to cooperate with your doctor to complete and evaluate the scale, including: cancer fatigue Scale, KPS score scale, quality of life scale.And daily on time to the hospital rehabilitation center outpatient clinic to receive acupuncture treatment.If you are a qualified participant, you can voluntarily participate in the study and sign an informed consent form.

(2) If you pass the above inspection, you will follow the following steps:

The study start will decide whether you will receive an acupuncture or aerobic exercise intervention based on the random numbers provided by the computer.The acupuncture group selected the acupoints of "Zusanli, Three Yin, Qi Hai, Guan Yuan, Baihui" for 30min for 21 days. The control group was the basic treatment group. The patients performed aerobic exercise in jogging mode, with the heart rate controlled at 50% of the highest heart rate (highest heart rate =220-patient age), exercising 5 days a week for 30min a day for 21 consecutive days.Patients are followed up 1 week after completion of treatment.Patients enrolled in this study had a 50% chance to be classified into these two different groups.Neither you nor your doctor was able to know or choose any of the intervention in advance.Study observations will last for 21 days.

Study day 1: You should go to the hospital, truthfully report the condition changes to the doctor, and fill in the self-evaluation form. The doctor will collect your medical history and physical examination results, and take free blood collection and feces as the research object.

You need to report recent physical symptom changes with your doctor during the study.

Study Day 21: Then the study ends.You should go to the hospital, the doctor will ask to record the changes of your condition, give you a physical examination, will fill in the scale again, and free blood collection, stool as the research object.

(3) Other matters

You need to come to the hospital at the agreed follow-up time between the doctor and you.Your follow-up is very important because the doctor will determine whether the study measures you receive are really working.

You will not receive other treatments for cancer fatigue, including medications, other psychotherapy treatments, etc.Please contact your doctor in advance.

A few people may have adverse reactions such as halo needle, pain, subcutaneous hematoma and so on during the trial and even lead to the termination of the trial.The reason is related to the personal response to acupuncture treatment; if the treatment has terminated the trial, we promise to do everything possible and give the best treatment.

**4.Potential benefits from participating in the research**

You and the society will likely benefit from this study.Such benefits include the potential for improvement in your condition and the possibility that this study may help develop a new treatment for other patients with similar conditions.

You will receive good medical care during the study period.

**5.Adverse reactions, risks, discomfort, and inconvenience of participating in the study**

Since the acupuncture treatment used in this study protocol is a routine treatment in traditional Chinese medicine, even if you do not participate in this clinical study, these adverse reactions may occur as long as you receive this treatment.The feeling of acid, numbness, weight and swelling appears in the process of acupuncture, which is the normal reaction of acupuncture.There may be adverse reactions after acupuncture, but less and mild, acupuncture may appear due to your physical problems or emotional tension, which can be relieved after stopping acupuncture and proper rest; bleeding and hematoma may occur after acupuncture, and can disappear after local compression: the doctor will strictly disinfect the operation during acupuncture.

If you have any discomfort during the study, or new changes in your condition, or any accidents, whether or not related to the treatment, timely notify your doctor and he / she will make judgment and medical treatment.The physician (or investigator) will do everything possible to prevent and treat injuries that may occur due to this study.

You need to come to the hospital on time for treatment and follow-up during the study, which may cause you trouble or inconvenience.

When filling in the cancer fatigue scale, please reflect objectively and truthfully, and choose the answer does not need to consider too long.

In addition, acupuncture treatment may be ineffective, and the disease continues to develop due to ineffective treatment or combined with other diseases.During the study period, if the acupuncture treatment measures taken in the study are ineffective, doctors will suspend the study and switch to other treatments that may be effective.

6. **Fees**

To compensate for the possible inconvenience you may participate in this study, this study will pay the acupuncture treatment done during this study, blood drawing, stool retention, and registration fee during follow-up.

If you also combine the treatment and examination required for other diseases, and the cost of switching to other treatments because the treatment fails, it will not be free.We will regularly observe the possible side effects / adverse effects caused by acupuncture treatment and take measures to prevent them.

**7.Confidentiality**

Your medical records (study medical records / CRF, laboratory sheets, etc.) will be kept intact in the hospital, and your doctor will record the laboratory test results on your outpatient medical records.The investigator, sponsor representatives, ethics committee and research supervision will be allowed access to your medical records.Any public report on the results of this study will not disclose your personal identity.We will make every effort to protect the privacy of your personal medical information as permitted by law.

Besides this study, it is possible that your medical records and pathology examination specimens will be used again in other future studies.You can also now declare denying studies other than this study utilizing your medical records and pathology specimens.

**8.More information**

You can ask any questions about this study at any time.Your doctor will leave you his / her phone number to answer your questions.

If you have any complaints about participating in the study, please contact the Ethics Committee Office.

Your doctor will inform you promptly if there is any important new information during the study that may affect your willingness to continue attending the study.

9. **You can voluntarily choose to participate in the study and withdraw from the study**

Whether to participate in the study depends entirely on your willingness.You may refuse to participate in this study or withdraw from this study at any time during the study, which will not affect the relationship with your doctor or the loss of your medical or other benefits.

Your doctor or investigator may suspend your participation in this study at any time in your best interest.

If you do not participate in this study, or withdraw from the study, there are many other alternative therapeutic drugs or methods.You do not have to choose to participate in this study in order to treat your illness.

If you withdraw from the study for any reason, you may be asked about your treatment.You may also be required to have a laboratory and physical examination if you believe so.This is very good for protecting your health.

10. **What should we do right now?**

It is up to you to participate in this study.You can discuss it with your family or friends before making a decision.

Before you make your decision to participate in the study, please ask your doctor any questions until you fully understand the study.

Thank you for reading the above material.If you decide to participate in this study, please tell your doctor or research assistant, and he / she will arrange everything for you about the study.

Please keep this information.

**Consent statement**

I have read the above introduction to this study and have the opportunity to discuss and ask questions with doctors about this study.All the questions I have asked have been satisfactorily answered.

I know the possible risks and benefits arising from participating in this study.I know that participation in the study is voluntary, and I confirm that I have enough time to consider this, and I understand that:

I can consult my doctor for more information.

I can withdraw from this study at any time without discrimination or retaliation, and my medical treatment and interests will not be affected.

I am also aware that if I quit the study, especially when I quit the study because of drugs, if I tell the doctor about the changes and complete the corresponding physical examination and physical and chemical examinations, it will be very beneficial to me and the whole study.

If I need any other medication due to the condition change, I will ask my doctor in advance or tell the doctor truthfully after the event.

I agree with the access to my research data by the research supervisor, the ethics committee or the sponsor representative.

I will obtain a signed and dated copy of the informed consent form.

Finally, I decided to agree to participate in this study.

Personal signature：	Date：

Telephone number ：	Cellphone number ：

Guardian signature：	Date：

Telephone number ：	Cellphone number ：

**The researcher's statement**

I confirm that the details of the trial, including its rights and possible benefits and risks, were explained to the subject and given a signed copy of the signed informed consent form.

Investigator signature：	Date：

Telephone number ：	Cellphone number ：

The Office of the Ethics Committee of the First Affiliated Hospital of Henan University of Chinese Medicine：0371-66285929

知情同意书·知情告知页

亲爱的受试者

您的医生已经诊断您患有癌因性疲乏疾病。

我们将邀请您参加一项基于“脑-肠轴”探讨针刺对乳腺癌癌因性疲乏的临床及机制研究的临床试验研究项目。本研究从中医针刺治疗入手，探讨针刺对乳腺癌化疗后CRF患者的疲乏情况、生存质量、体力状况等临床疗效评价，同时基于肠道菌群对HPA轴的影响进而探讨针刺对乳腺癌化疗后CRF的作用机制研究。在您决定是否参加这项研究之前，请尽可能仔细阅读以下内容，它可以帮助您了解该项研究以及为何要进行这项研究，研究的程序和期限，参加研究后可能给您带来的益处、风险和不适。如果您愿意，您也可以和您的亲属、朋友一起讨论，或者请您的医生给予解释，帮助您做出决定。

研究介绍

1. 为什么要开展本项研究？

癌因性疲乏（Cancer-Related Fatigue, CRF）是肿瘤患者以主观感觉疲乏为主诉的一组症状，由Haylock及Hart两位学者首次提出。肿瘤患者的疲乏是一种典型的身体和心理共同疾病，它不但使身体感觉力量的缺乏，还影响患者的内心情感世界和对外界的认知反应，严重降低患者的生活质量，增加了对疾病和治疗的惧怕心理，削弱了依从性，甚至迫使患者无故拒绝接受治疗。这种疾病使患者身心受创，即使经过睡觉、放松、休息等舒缓疲乏的常见措施处理，症状仍无法得到缓解。NCCN指南将CRF定义为“一种与肿瘤或肿瘤治疗相关，痛苦、持续的包含生理、情感和认知层面上的主观疲劳感”。该病在肿瘤患者中非常常见，可以发生于肿瘤病程的任何阶段。据统计，约82%-96%接受放疗的患者会发生CRF，接受化疗的患者比例为65%-100%。目前临床上治疗CRF分两类：一是药物性干预，包括中枢兴奋剂；二是非药物性干预，包括体力活动、心理社会干预等。这些措施多属于对症处理，总体疗效差强人意。

鉴于此，积极探索新方法，研究最优的治疗方案为目前治疗癌因性疲乏亟待解决的关键所在。故本研究从中医针灸疗法分析，通过刺激人体经络穴位调节人体气血、改善人体生理功能从而减轻或消除乳腺癌患者的疲劳状态，全面改善患者身体状况，提高患者生存质量，并寻找其起效机制。

本研究将在河南中医药大学第一附属医院康复中心门诊进行，预计本单位有70名乳腺癌化疗后癌因性疲乏受试者自愿参加。

本项研究已经得到研究管理部门同意。河南中医药大学第一附属医院伦理委员会已经审议此项研究是遵从赫尔辛基宣言原则，符合医疗道德的。

二、哪些人适合参加研究

本研究受试人群的纳入标准为：（1）符合ICD-10癌因性疲乏诊断标准；；（2）经病理或细胞学诊断的乳腺癌患者；（3）年龄18-80岁；（4）一般情况可，KPS评分＞50，预计生存期半年及以上；（5）之前行化疗治疗，并在接下来21天内无其他治疗计划；（6）无精神障碍及认知障碍，有正常语言表达能力，能理解量表内容并配合治疗；（7）无严重的心、肝、肾基础疾病；（8）自愿参加研究并签署知情同意书者。

排除标准：（1）有严重的基础疾病包括血液、心血管、肝、肾疾病、严重感染等；（2）诊断明确的精神或心理疾病，包括认知障碍、焦虑抑郁证等；（3）妊娠期或哺乳期女性；（4）语言沟通障碍或其他原因无法配合随访及检查；（5）晕针患者。

三、如果参加研究将需要做什么

1. 在您入选研究前，您将接受以下检查以确定您是否可以参加研究：

医生将询问、记录您的病史，对您进行全面的体格和心理检查。您需要配合医生完成量表的填写和评估，包括：癌因性疲乏量表、KPS评分表、生活质量量表。并且每日按时到医院康复中心门诊接受针刺治疗。若您是合格的纳入者，您可自愿参加研究，签署知情同意书。

2. 若您以上检查合格，将按以下步骤进行研究

研究开始将根据计算机提供的随机数字，决定您接受针刺或有氧运动干预措施。针刺组选取“足三里、三阴交、气海、关元、百会”穴位，一周治疗5次，每次30min，共治疗21天；对照组为基础治疗组，患者进行慢跑模式的有氧运动锻炼，其中心率控制在最高心率的50%（最高心率=220-患者年龄），一周锻炼5天，每天30min，共连续进行21天。治疗结束后1周随访。参加这项研究的患者分别有50%的可能性被分入这两个不同的组别。您和您的医生都无法事先知道和选择任何一种干预措施。研究观察将持续21天。

研究第1天：您应到医院就诊，并如实向医生反映病情变化，填写自评量表，医生将收集您的病史及体检结果，同时免费采血、粪便一次作为研究对象。

研究期间您需与医生反映近期身体症状变化。

研究第21天：这时候研究结束了。您应到医院就诊，医生将询问记录您病情的变化，给您做体格检查，还将再次填写量表，并免费采血、粪便一次作为研究对象。

3. 需要您配合的其他事项

您需要按医生和您约定的随访时间来医院就诊。您的随访非常重要，因为医生将判断您接受的研究措施是否真正起作用。

在研究期间您不能接受治疗癌因性疲乏的其他疗法，包括药物疗法、其他心理治疗等。如您需要进行其他治疗，请事先与您的医生取得联系。

极少数人有可能在试验过程中出现晕针、疼痛、皮下血肿等不良反应甚至导致试验终止。其原因和个人对针刺治疗的反应有关；如果出现了和治疗相关的导致试验终止的情况，我们承诺：必将全力以赴，给予最好的治疗。

关于饮食、生活起居的规定：少食辛辣、油腻。

四、参加研究可能的受益

您和社会将可能从本项研究中受益。此种受益包括您的病情有可能获得改善，以及本项研究可能帮助开发出一种新治疗方法，以用于患有相似病情的其他病人。

您将在研究期间获得良好的医疗服务。

五、参加研究可能的不良反应、风险和不适、不方便

由于本研究方案中使用的针刺治疗属于中医常规治疗，即使您不参加本临床研究，只要接受该治疗方法，就有可能发生这些不良反应。针刺过程中出现的酸、麻、重、胀的感觉，这均为针刺的正常反应。针刺后可能存在不良反应，但较少而轻微，针刺时可能因为您的体质问题或情绪紧张出现晕针现象，经停止针刺和适当休息后可缓解；针刺后可能出现出血、血肿等现象，经局部按压后可消失：针刺过程中医生会严格消毒操作。

如果您在研究中出现任何不适，或病情发生新的变化，或任何意外情况，不管是否与治疗方法有关，均应及时通知您的医生，他/她将对此作出判断和医疗处理。医生（或研究者）将尽全力预防和治疗由于本研究可能带来的伤害。

您在研究期间需要按时到医院治疗和随访，这些都有可能给您造成麻烦或带来不便。

在填写癌因性疲乏量表时请客观如实反映，选择答案不需考虑太久。

此外，针刺治疗可能出现无效的情况，以及因治疗无效或者因合并其他疾病等原因而导致病情继续发展。在研究期间，如果医生发现本项研究所采取的针刺治疗措施无效，将会中止研究，改用其他可能有效的治疗措施。

1. 有关费用

为了补偿您参加本研究可能给您带来的不便，本研究将支付您参加本项研究期间所做的针刺治疗费用、抽血、留取粪便费用以及随访时的挂号费。

如果您同时合并其他疾病所需的治疗和检查，以及因治疗无效而改用其他治疗的费用，将不在免费的范围之内。我们将通过定期观察针刺治疗可能引起的副作用/不良反应，并采取措施加以防治。

七、个人信息是保密的吗？

您的医疗记录（研究病历/CRF、化验单等）将完整地保存在医院，医生会将化验检查结果记录在您的门诊病历上。研究者、申办者代表、伦理委员会和研究监督管理部门将被允许查阅您的医疗记录。任何有关本项研究结果的公开报告将不会披露您的个人身份。我们将在法律允许的范围内，尽一切努力保护您个人医疗资料的隐私。

除本研究以外，有可能在今后的其他研究中会再次利用您的医疗记录和病理检查标本。您现在也可以声明拒绝除本研究外的其他研究利用您的医疗记录和病理标本。

八、怎样获得更多的信息？

您可以在任何时间提出有关本项研究的任何问题。您的医生将给您留下他/她的电话号码以便能回答您的问题。

如果您对参加研究有任何抱怨，请联系伦理委员会办公室。

如果在研究过程中有任何重要的新信息，可能影响您继续参加研究的意愿时，您的医生将会及时通知您。

九、可以自愿选择参加研究和中途退出研究

是否参加研究完全取决于您的自愿。您可以拒绝参加此项研究，或在研究过程中的任何时间退出本研究，这都不会影响您和医生间的关系，都不会影响对您的医疗或有其他方面利益的损失。

您的医生或研究者出于对您的最大利益考虑，可能会随时中止您参加本项研究。

如果您不参加本项研究，或中途退出研究，还有很多其它可替代的治疗药物或者方法。您不必为了治疗您的疾病而必须选择参加本项研究。

如果您因为任何原因从研究中退出，您可能被询问有关您治疗的情况。如果医生认为需要，您也可能被要求进行实验室检查和体格检查。这对保护您的健康十分有利。

十、现在该做什么？

是否参加本项研究由您自己决定。您可以和您的家人或者朋友讨论后再做出决定。

在您做出参加研究的决定前，请尽可能向您的医生询问有关问题，直至您对本项研究完全理解。

感谢您阅读以上材料。如果您决定参加本项研究，请告诉您的医生或研究助理，他/她会为您安排一切有关研究的事务。

请您保留这份资料。

知情同意书·同意签字页

受试者同意声明

我已经阅读了上述有关本研究的介绍，而且有机会就此项研究与医生讨论并提出问题。我提出的所有问题都得到了满意的答复。

我知道参加本研究可能产生的风险和受益。我知晓参加研究是自愿的，我确认已有充足时间对此进行考虑，而且明白：

- 我可以随时向医生咨询更多的信息。
- 我可以随时退出本研究，而不会受到歧视或报复，医疗待遇与权益不会受到影响。

我同样清楚，如果我中途退出研究，特别是由于药物的原因使我退出研究时，我若将病情变化告诉医生，完成相应的体格检查和理化检查，这将对我本人和整个研究十分有利。

如果因病情变化我需要采取任何其他的药物治疗，我会在事先征求医生的意见，或在事后如实告诉医生。

我同意研究监督管理部门、伦理委员会或申办者代表查阅我的研究资料。

我将获得一份经过签名并注明日期的知情同意书副本。

最后，我决定同意参加本项研究。

受试者签名： 　＿ ＿ ＿ ＿ 年 ＿ ＿ 月 ＿ ＿ 日

受试者联系电话：	手机号：

受试者法定监护人签名：	＿ ＿ ＿ ＿ 年 ＿ ＿ 月 ＿ ＿ 日

法定监护人联系电话：	手机号：

研究者声明

我确认已向受试者解释了本试验的详细情况，包括其权利以及可能的受益和风险，并给其一份签署过的知情同意书副本。

研究者签名：	日期：＿ ＿ ＿ ＿ 年 ＿ ＿ 月 ＿ ＿ 日

研究者的工作电话：	手机号：

河南中医药大学第一附属医院伦理委员会办公室联系电话：0371-66285929
